# Vascular risk factors and neuroimaging heterogeneity across different white matter hyperintensities distribution patterns

**DOI:** 10.3389/fnhum.2025.1633355

**Published:** 2025-07-28

**Authors:** Junjun Wang, Liying Zhuang, Fengli Fu, Yejia Mo, Lihao Zhai, Qi Xu, Caiyun Mou

**Affiliations:** ^1^Department of Neurology, Zhejiang Hospital, Hangzhou, China; ^2^Department of Radiology, Zhejiang Hospital, Hangzhou, China

**Keywords:** white matter hyperintensities, vascular risk factors, white matter microstructural injury, cerebral blood flow, heterogeneity, cognitive impairment

## Abstract

**Background:**

Different white matter hyperintensities (WMHs) distribution patterns exhibit distinct clinical implications, but their underlying mechanisms remain unclear. This study explores vascular risk factors and neuroimaging features to elucidate their heterogeneity.

**Methods:**

We retrospectively analyzed WMHs patients who underwent multimodal MRI at Zhejiang Hospital. Neuroimaging features included gray matter volume, white matter microstructure (Fractional anisotropy, FA), and cerebral blood flow (CBF) were assessed. Vascular risk factors and imaging features were compared across four different WMHs distribution patterns [multi-spots, peri-basal ganglia, anterior subcortical (SC) patches, and posterior SC patches]. Mediation analysis was performed to explore the role of imaging features on WMHs related cognitive impairment.

**Results:**

A total of 163 patients were included in the final analysis. Among the four WMHs distribution patterns, hypertension was significantly more prevalent in patients with anterior SC patches [48 [85.7%] vs. 71 [66.4%], *p* = 0.008]. All WMH distribution patterns except multi-spots exhibited reduced gray matter volume (Bonferroni *p* < 0.0125). Notably, only patients with anterior SC patches exhibited a reduction in white matter FA (0.342 ± 0.049 vs. 0.370 ± 0.043, *p* < 0.001). Furthermore, patients with posterior SC patches displayed significantly lower CBF in both gray matter (42.65 ± 11.76 vs. 48.02 ± 10.97, *p* = 0.003) and white matter (35.25 ± 8.81 vs. 38.86 ± 8.07, *p* = 0.007). Mediation analysis revealed that white matter microstructural injury mediated the association between anterior SC patches WMHs and cognitive impairment [β = −0.371, Bootstrap 95% CI [−0.939, −0.006]].

**Conclusion:**

This study demonstrates heterogeneity in vascular risk factors, gray matter volume, microstructural injury, and hypoperfusion across different WMHs patterns, underscoring the importance of subtype-specific mechanistic and therapeutic research.

## Introduction

White matter hyperintensities (WMHs), a neuroimaging hallmark of cerebral small vessel disease (CSVD), appear as hyperintense lesions on T2-weighted or fluid attenuated inversion recovery (FLAIR) MRI sequences (Duering et al., 2023). These lesions are highly prevalent in the aging population, affecting up to 80% of healthy individuals aged 60 and older (de Leeuw et al., 2001). Literatures have demonstrated that greater WMH volumes correlate with a higher likelihood of late-onset cognitive impairment and dementia (Puzo et al., 2019), and their severity may influence the risk and progression of Alzheimer's disease (AD) (Eloyan et al., 2023). Furthermore, emerging evidence indicates a potential association between WMHs and both gait abnormalities and affective disorders (Crook et al., 2020; Pearcy et al., 2025). Although WMHs are thought to arise from blood-brain barrier disruption, impaired cerebral blood flow (CBF) regulation, and subsequent white matter demyelination, their precise pathophysiological mechanisms remain unclear, limiting the development of effective therapeutic interventions (Wardlaw et al., 2019).

As is well-established, WMHs exhibit characteristic spatial distribution patterns, typically occurring along periventricular or deep subcortical regions (Fernando et al., 2006). Previous studies have demonstrated an association between periventricular WMHs and cognitive function (Jung Hwa et al., 2011). However, some investigations have suggested that periventricular WMHs may be more specifically linked to gait abnormalities, whereas subcortical WMHs appear to be associated with cognitive decline (Silbert et al., 2008). These findings imply that WMHs in different regions may contribute to distinct clinical implications, although significant heterogeneity exists among these studies (Bolandzadeh et al., 2012; Shim et al., 2015). To address this variability, Charidimou et al. classified WMHs into four subtypes based on distribution patterns, demonstrating that WMHs multiple subcortical spots were associated with cerebral amyloid angiopathy-related hemorrhage, whereas peri-basal ganglia WMHs correlated with hypertensive hemorrhage (Charidimou et al., 2016). Subsequent studies further revealed that WMHs with anterior subcortical patches pattern were strongly linked to all-cause dementia (Wang et al., 2022). Similarly, some studies found that deep frontal WMHs, a distribution resembling anterior subcortical patches pattern, were also associated with cognitive impairment (Habes et al., 2018; Phuah et al., 2022).

Building on these literatures, the distinct clinical implications of WMH distribution patterns may reflect underlying neuropathological heterogeneity (Kim et al., 2008; Shim et al., 2015). Previous neuroimaging studies have demonstrated that frontal and parietal periventricular WMHs, along with deep WMHs, are more extensive in AD patients compared to cognitively normal elderly individuals (Polvikoski et al., 2010). The Baltimore Longitudinal Study further revealed that frontal WMHs emerge as early as the fifth decade of life, showing significant associations with both systolic blood pressure (Habes et al., 2018). While these findings suggest region-specific pathological mechanisms for WMHs, current evidence primarily focuses on vascular risk factors such as age and hypertension. Crucially, the role of myelin degeneration and cerebral hypoperfusion across distinct WMH regions remain poorly understood.

Neuroimaging techniques, particularly diffusion tensor imaging (DTI), allow for the evaluation of white matter demyelination by measuring fractional anisotropy (FA) (Chang et al., 2017). A decrease in FA values indicates white matter (WM) microstructural injury, which aligns with histopathological features such as elevated water content, myelin loss, and axonal injury (Filler, 2009). Previous studies have demonstrated FA reductions in both WMHs and normal-appearing WM in patients with CSVD (Pasi et al., 2016). Furthermore, Min et al. reported regional variations in FA values across periventricular WMHs in different brain lobes (Min et al., 2021). Additionally, arterial spin labeling (ASL) provides a non-invasive measure of CBF, with prior evidence linking hypoperfusion to WMH progression (Faezeh et al., 2025). However, existing research on WM microstructure and CBF in WMHs has primarily focused on global WMHs or specific fiber tracts, with limited attention to different WMHs distribution patterns. To address this gap, our study employs multimodal MRI to investigate the heterogeneity of vascular risk factors and neuroimaging features across four different WMH distribution patterns, thereby elucidating potential mechanisms underlying their differential effects on neural function.

## Materials and methods

### Subjects

We conducted a retrospective analysis of patients with WMHs who underwent multimodal MRI scanning at Zhejiang Hospital between January 2021 and December 2024. The inclusion criteria were: (1) age ≥40 years; (2) presence of WMH on T2 FLAIR MRI; (3) availability of 3D-T1, T2 FLAIR, DTI, and ASL sequences; and (4) underwent Montreal Cognitive Assessment (MoCA) evaluation. Exclusion criteria comprised: (1) cerebral infarction (except for non-acute lacunar infarcts), intracranial hemorrhage, brain tumors, traumatic brain injury, or encephalitis; (2) secondary white matter lesions due to metabolic, toxic and other causes; and (3) poor image quality precluding analysis. We retrieved patients' demographic characteristics, vascular risk factors, and imaging data, such as age, sex, education duration, MoCA scores, hypertension, diabetes mellitus, hyperlipidemia, smoking history and so on.

### MRI protocol

MRI scanning was performed on a 3-Tesla MR scanner (Skyra, Siemens, Germany) with a 20-channel phased array head coil. The parameters of the MR sequences were as follows: 3D T1 Weighted Imaging Magnetization Prepared Rapid Gradient Echo (3D T1WI MPRAGE): repetition time 2,200 ms, echo time 2.5 ms, inversion time 900 ms, field of view 256 × 256 mm^2^, matrix size 256 × 256, slice thickness 1 mm; T2 FLAIR imaging: repetition time 9,000 ms, echo time 95 ms, inversion time 2,500 ms, field of view 256 × 256 mm^2^, matrix size 256 × 256, slice thickness 2 mm; DTI: repetition time 8,000 ms, echo time 89 ms, field of view 256 × 256 mm^2^, matrix size 128 × 128, resolution 1 × 1 × 2 mm, slice thickness 2 mm, 30 non-collinear directions, maximum b-value 1,000 s/mm^2^; ASL were acquired using three-dimensional pseudo-continuous ASL sequence: repetition time 4,600 ms, echo time 15.8 ms, field of view 144 × 144 mm^2^, matrix size 64 × 64, slice thickness 4 mm.

### Assessment of WMHs

**Figure 1:**
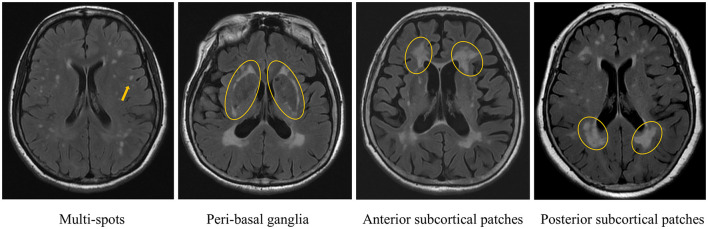
Different white matter hyperintensities (WMHs) distribution patterns.

WMHs was defined as non-cavitary hyperintense lesions on T2 FLAIR, following the Standards for Reporting Vascular Changes on Neuroimaging (Wardlaw et al., 2013). WMHs patterns were evaluated using RadiAnt DICOM Viewer (v2024.1). Raw imaging data were imported into the software, and four distinct WMH patterns were independently assessed on axial T2 FLAIR sequences, following the methodology outlined in prior research (Charidimou et al., 2016) (Figure 1): (1) multi-spots pattern: scattered small circular WMHs (>10 lesions) in subcortical white matter; (2) peri- basal ganglia (BG) pattern: WMHs conforming to the outer contours of the basal ganglia; (3) anterior subcortical (SC) patches pattern: large confluent WMHs (>5 mm extension) in deep white matter, anterior to the frontal horn/ventricular body junction, distinctly separate from periventricular WMHs; and (4) posterior SC patches pattern: similarly extensive WMHs (>5 mm) posterior to the ventricular horn, with clear demarcation from periventricular involvement. Two trained raters (Wang. J. and Mou. C., with 10and 14 years of neuroimaging review experience, respectively) independently classified all cases. Discrepancies were resolved through consensus. Intra-rater reliability was assessed by Wang. J., who re-evaluated 50 randomly selected cases after a 3-month interval.

### Evaluation of gray matter volume, white matter microstructural injury, and cerebral perfusion

**Figure 2:**
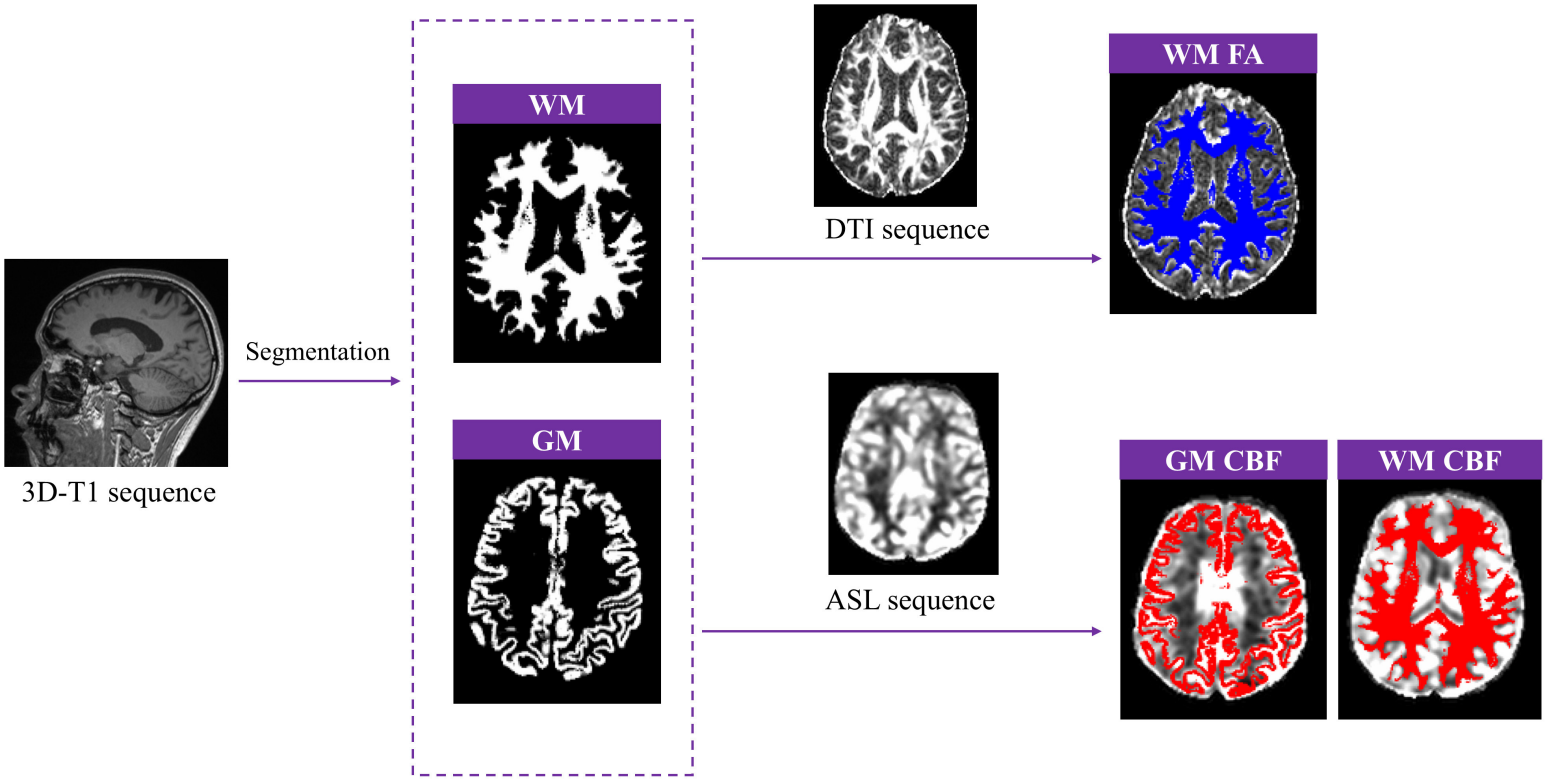
Post-processing of gray matter (GM) volume, white matter fractional anisotropy (WM FA), GM and WM cerebral blood flow (CBF).

First, the raw 3D-T1 images were converted to the NIFTI (.nii.gz) format using MRIcroGL software. Subsequently, SPM12 (Statistical Parametric Mapping 12; www.fil.ion.ucl.ac.uk/spm/) was implemented in MATLAB (Version 2019a; www.mathworks.com) to perform tissue segmentation. The “Segment” tool in SPM12 was used to process the 3D-T1 images, generating separate whole-brain WM and gray matter (GM) maps, and calculating the volume of GM. DTI images were post-processed using FSL (www.fmrib.ox.ac.uk/fsl), which involved brain extract, the removal of bulk motion artifacts, and the correction of eddy current-induced distortions. FA values were then calculated using DTIfit command in FSL. ASL raw data were transferred to the workstation of Siemens MR, where quantitative CBF maps were generated using a custom-built program. The WM mask was co-registered with the FA images, while the WM and GM masks were co-registered with the CBF maps. This process resulted in the WM FA map, WM CBF map, and GM CBF map (Figure 2). Finally, the corresponding FA and CBF values were extracted using MATLAB software.

### Statistical analysis

All statistical analyses were performed using SPSS 22.0 (IBM Corp.). Continuous variables with normal distribution were compared between groups using independent samples *t*-tests, while non-normally distributed variables were analyzed using non-parametric tests. For multiple comparisons across groups, the Bonferroni correction was applied. Mediation analysis was conducted using the PROCESS (v4.0) in SPSS, with WMHs pattern as the independent variable, imaging features as the mediator, and MoCA score as the dependent variable. Bootstrap resampling (*n* = 5,000) was performed to assess mediation effects, with statistical significance determined if both the total effect and indirect (mediated) effect were significant. A two-tailed *p*-value < 0.05 was considered statistically significant. Given the four WMHs pattern categories, a Bonferroni-adjusted threshold of *p* < 0.0125 was applied for multiple comparisons.

## Results

### Patient characteristics

**Figure 3:**
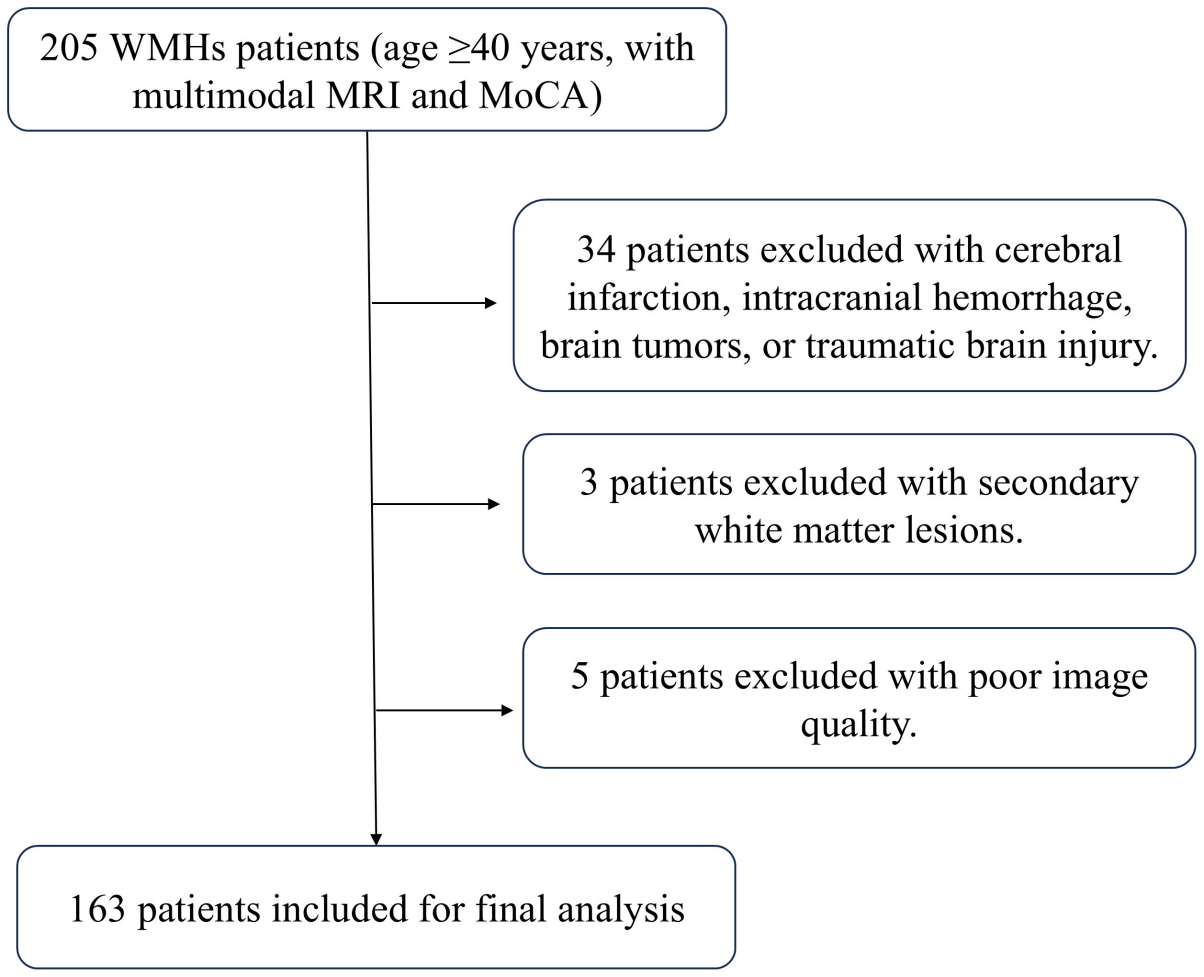
Flow chart of the selection process of the white matter hyperintensities (WMHs) patients.

A total of 163 patients were ultimately enrolled for final analysis. Figure 3 shows the flow chat. Among the patients, multi-spots were observed in 124 cases (76.1%), peri-BG in 33 cases (20.2%), anterior SC patches in 56 cases (34.4%), and posterior SC patches in 84 cases (51.5%). The inter-rater intraclass correlation coefficients (ICCs) were 0.917 for multi-spots, 0.878 for peri-BG, 0.915 for anterior SC patches and 0.840 for posterior SC patches. The intra-rater ICCs were 0.918 for multi-spots, 0.878 for peri-BG, 0.913 for anterior SC patches and 0.880 for posterior SC patches. Details are presented in Table 1.

**Table 1:** Clinical and demographic data of patients.

**Characteristics**	***n* = 163**
Age, years old, mean ± sd	67.0 ± 10.2
Female, *n* (%)	76 (46.6)
Years of education, median (IQR)	8 (3, 12)
MoCA, mean ± sd	21.4 ± 5.3
Hypertension, *n* (%)	119 (73.0)
Diabetes, *n* (%)	31 (19.0)
Hyperlipoidemia, *n* (%)	25 (15.3)
Smoking, *n* (%)	32 (19.6)
Multi-spots pattern, *n* (%)	124 (76.1)
Peri-BG pattern, *n* (%)	33 (20.2)
Anterior SC patches pattern, *n* (%)	56 (34.4)
Posterior SC patches pattern, *n* (%)	84 (51.5)
Gray matter volume, mL, mean ± sd	610.29 ± 76.60
White matter FA, mean ± sd	0.360 ± 0.047
Gray matter CBF, mL/100 g/min, mean ± sd	45.25 ± 11.67
White matter CBF, mL/100 g/min, mean ± sd	37.00 ± 8.63

IQR, interquartile range; BG, basal ganglia; SC, subcortical; FA, fractional anisotropy; CBF, cerebral blood flow.

### Association between different WMH patterns and vascular risk factors

After multiple comparison correction (Bonferroni *p* < 0.0125), patients with anterior SC patches (70.4 ± 8.9 vs. 65.3 ± 10.4, *p* = 0.002) or posterior SC patches (69.0 ± 9.9 vs. 65.0 ± 10.2, *p* = 0.011) were significantly older than those without these WMH patterns. Moreover, hypertension was more prevalent in patients with anterior SC patches [48 [85.7%] vs. 71 [66.4%], *p* = 0.008] across these four WMHs patterns, Table 2.

**Table 2:** Association between different WMH patterns and vascular risk factors.

**Variables**	**Multi-spots**			**Peri-BG**			**Anterior SC patches**			**Posterior SC patches**		
	**Yes**, ***n*** = **124**	**No**, ***n*** = **139**	***p-*** **value**	**Yes**, ***n*** = **33**	**No**, ***n*** = **130**	***p-*** **value**	**Yes**, ***n*** = **56**	**No**, ***n*** = **107**	***p-*** **value**	**Yes**, ***n*** = **84**	**No**, ***n*** = **79**	***p-*** **value**
Age, mean ± sd	66.8± 10.2	67.9± 10.4	0.556	68.6 ± 9.5	66.6 ± 10.4	0.312	70.4 ± 8.9	65.3 ± 10.4	0.002^*^	69.0 ± 9.9	65.0 ± 10.2	0.011^*^
Female, *n* (%)	57 (46.0)	19 (48.7)	0.764	17 (51.5)	59 (45.4)	0.528	27 (48.2)	49 (45.8)	0.769	33 (39.3)	43 (54.4)	0.053
Hypertension, *n* (%)	89 (71.8)	30 (76.9)	0.528	26 (78.8)	93 (71.5)	0.402	48 (85.7)	71 (66.4)	0.008^*^	64 (76.2)	55 (69.6)	0.345
Diabetes, *n* (%)	19 (15.3)	12 (30.8)	0.032	3 (9.1)	28 (21.5)	0.104	8 (14.3)	23 (21.5)	0.265	17 (20.2)	14 (17.7)	0.682
Hyperlipoidemia, *n* (%)	19 (15.3)	5 (12.8)	0.701	3 (9.1)	21 (16.2)	0.307	11 (19.6)	13 (12.1)	0.200	18 (21.4)	6 (7.6)	0.013
Smoking, *n* (%)	25 (20.2)	7 (17.9)	0.762	5 (15.2)	27 (20.8)	0.468	9 (16.1)	23 (21.5)	0.408	17 (20.2)	15 (19.0)	0.841

WMH, white matter hyperintensities; BG, basal ganglia; SC, subcortical.

^*^p < 0.0125 after Bonferroni correction.

### Heterogeneity in neuroimaging features across different WMH patterns

**Figure 4:**
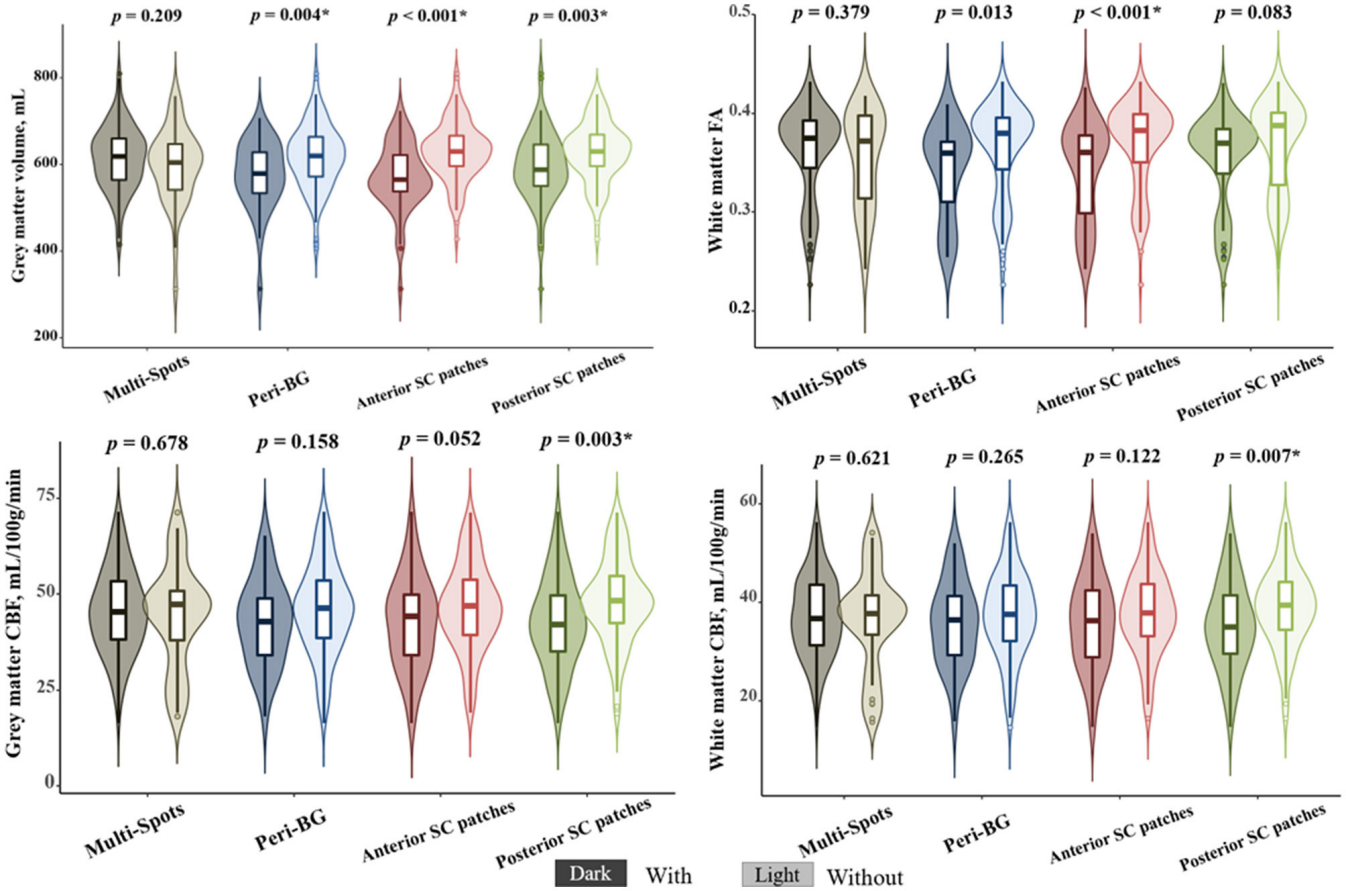
The correlations between different white matter hyperintensities (WMHs) patterns and neuroimaging features. *Bonferroni *p* < 0.0125.

As shown in Figure 4, after multiple comparison correction (Bonferroni *p* < 0.0125), patients with peri-BG (575.42 ± 81.68 vs. 618.81 ± 73.13, *p* = 0.004), anterior SC patches (571.04 ± 77.26 vs. 630.84 ± 68.04, *p* < 0.001), and posterior SC patches (593.18 ± 80.91 vs. 628.49 ± 67.59, *p* = 0.003) exhibited significantly lower gray matter volume compared to those without these WMH patterns, while no significant difference was observed in the multi-spots pattern. Among these four distribution patterns, only patients with anterior SC patches showed reduced white matter FA (0.342 ± 0.049 vs. 0.370 ± 0.043, *p* < 0.001). Additionally, those with posterior SC patches demonstrated significantly decreased GM CBF (42.65 ± 11.76 vs. 48.02 ± 10.97, *p* = 0.003) and WM CBF (35.25 ± 8.81 vs. 38.86 ± 8.07, *p* = 0.007) compared to patients without this pattern. Multivariate regression analysis incorporating the different WMH patterns showed that, after adjusting for age, anterior SC patches WMH significantly correlated with gray matter volume (β = −40.143, *p* = 0.003) and white matter FA (β = −0.023, *p* = 0.011, while posterior SC patches WMH was associated with gray CBF (β = −4.060, *p* = 0.047) and white matter CBF (β = −3.176, *p* = 0.042), Table 3.

**Table 3:** Multivariate regression analysis of the different WMH patterns after age adjustment.

**WMH patterns**	**Gray matter volume**		**White matter FA**		**Gray matter CBF**		**White matter CBF**	
	β	***p***	β	***p***	β	***p***	β	***p***
Multi-spots	21.610	0.091	0.010	0.260	1.470	0.485	1.383	0.390
Peri-BG	−14.141	0.331	−0.010	0.328	−0.934	0.698	−0.190	0.918
Anterior SC patches	−40.143	0.003^*^	−0.023	0.011^*^	0.354	0.871	0.276	0.868
Posterior SC patches	−7.063	0.565	−0.001	0.996	−4.060	0.047^*^	−3.176	0.042^*^

WMH, white matter hyperintensities; FA, fractional anisotropy; CBF, cerebral blood flow; BG, basal ganglia; SC, subcortical.

^*^p < 0.05.

### Mediating effect of imaging features on the association between WMH patterns and MoCA scores

**Figure 5:**
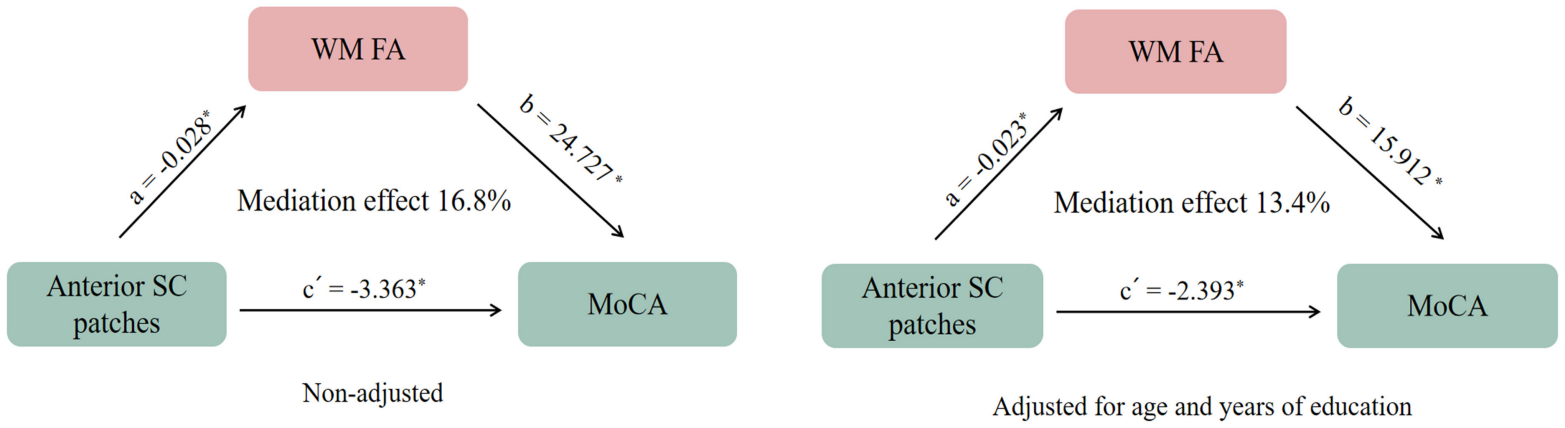
The mediating effect of white matter microstructural fractional anisotropy (WM FA) on the association between (white matter hyperintensities) WMHs patterns and Montreal Cognitive Assessment (MoCA) Scores. *p < 0.05.

In this study, the mean MoCA score was 21.4 ± 5.3. As shown in Supplementary Table 1, among the four WMH patterns, patients with anterior SC patches exhibited significantly lower MoCA scores compared to those without this pattern (19.7 ± 5.7 vs. 22.7 ± 5.0, *p* = 0.001). Mediation analysis revealed that WM FA significantly mediated the association between anterior SC patches and MoCA scores [β = −0.679, Bootstrap 95% CI [−1.371, −0.156], total effect = −4.042, direct effect = −3.363, and indirect effect = −0.679]. Similarly, GM volume also significantly mediated this association [β = −0.772, Bootstrap 95% CI [−1.481, −0.162], total effect = −4.042, direct effect = −3.270, and indirect effect = −0.772]. After adjusting for age and years of education, the mediating effect of WM FA remained significant [β = −0.371, Bootstrap 95% CI [−0.939, −0.006], total effect = −2.764, direct effect = −2.393, and indirect effect = −0.371], whereas GM volume no longer showed a significant mediating effect [β = −0.182, Bootstrap 95% CI [−0.722, 0.272], total effect = −2.764, direct effect = −2.582, and indirect effect = −0.182], Figure 5.

## Discussion

Our study revealed hypertension emerged as specific risk factors for anterior SC patches. Compared to other WMHs patterns, anterior SC patches demonstrated more severe white matter microstructural damage, while posterior SC patches showed more pronounced CBF reduction. Furthermore, the cognitive impairment associated with anterior SC patches WMHs was partially mediated by WM microstructural injury.

These distribution-specific differences align with prior neuroanatomical studies demonstrating distinct vascular vulnerabilities across brain regions. Studies focusing on localized rather than total WMH burden have consistently identified frontal WMHs as being strongly linked to hypertension (Newton et al., 2023; Phuah et al., 2022). This frontal predominance shows remarkable anatomical concordance with our findings of hypertension-associated WMHs concentrated in anterior subcortical regions. The anterior SC patches, primarily supplied by penetrating arteries from the anterior circulation, may be more susceptible to hypertensive arteriopathy. Moreover, neuropathological evidence from postmortem tissue demonstrates that arteriolosclerosis predominantly drives frontal WMHs, consistent with our identified pattern of anterior SC patch involvement (McAleese et al., 2017). Our findings may potentially offer some preliminary insights for future research on blood pressure management in ameliorating WMHs.

Our study identified a strong correlation between anterior SC patches WMHs and WM microstructural injury, potentially attributable to the high prevalence of hypertension in this subgroup. Existing evidence indicates that hypertension induces WM microstructural injury (Junyeon et al., 2024), with preferential vulnerability observed in the long-range fibers traversing anterior frontal lobes (Zilin et al., 2023). This finding aligns with previous studies. A CSVD study has identified significant correlations between frontal WMHs and advanced diffusion markers of white matter integrity, such as peak width of skeletonized mean diffusivity (Low et al., 2020). Prior study suggested lower FA values were associated with WMHs observed in the periventricular frontal lobe, adjacent to anterior SC patches (Min et al., 2021). The particular sensitivity of these regions may stem from their unique neuroanatomical architecture. DTI studies reveal that anterior subcortical areas serve as critical convergence zones for multiple major fiber pathways, including the fronto-occipital fasciculus, cingulum bundle, callosal radiations, and superior longitudinal fasciculus (Wandell, 2016). This may explain the strong association observed between anterior SC patches WMHs and WM microstructural injury. While these findings provide important anatomical context, the precise pathophysiological pathways require further elucidation.

The pronounced CBF reduction in patients with posterior SC patches WMHs likely reflects their unique vascular architecture. As watershed zones between the middle cerebral artery and posterior cerebral artery territories, these regions are particularly vulnerable to hypoperfusion. In patients with hypoxic-ischemic encephalopathy, the posterior watershed zones are particularly vulnerable to WM injury (Cao et al., 2023). Hypoperfusion disrupts white matter integrity by inducing the loss of oligodendrocyte, thus myelin, and axonal structure and function. However, the cross-sectional design limits causal inferences regarding WMH progression, and future longitudinal studies should validate these subtype-specific trajectories.

Our study further revealed that the impact of anterior SC patches WMHs on cognition is partially mediated by white matter microstructural injury. In patients with CSVD, such microstructural injury has been consistently associated with slower processing speed and poorer working memory (Dao et al., 2021). These microstructural disruptions compromise efficient neuronal signal transmission and disrupt functional connectivity across distributed brain networks (Combes et al., 2022). Notably, emerging evidence suggests that WM microstructural abnormalities may also be linked to β-amyloid accumulation (Pietroboni et al., 2019), potentially mediated by the blood-brain barrier dysfunction. These findings underscore the importance of evaluating not only visible WMHs but also covert microstructural injury in WMHs patients.

A key strength of our study lies in the application of multimodal neuroimaging to systematically analyze the heterogeneity of imaging characteristics and vascular risk factors across distinct WMHs patterns. Our findings not only provide insights into potential subtype-specific pathological mechanisms, but also paves the way for developing personalized therapeutic strategies tailored to individual WMHs patterns.

This study has several limitations that should be acknowledged. First, as the participants were hospital-based patients rather than community-dwelling individuals, the observed WMHs burden may represent more severe cases than those found in the general population. Second, the relatively small sample size and cross-sectional design may introduce potential biases and cannot establish causality for the observed relationships. Third, our CBF measurements were based on single post-labeling delay ASL, which may underestimate CBF due to transit time and watershed artifacts. These preliminary observations warrant verification through larger cohort studies with longitudinal follow-up.

## Conclusion

Our study reveals regional heterogeneity in vascular risk factors, GM volume, WM microstructural injury, and hypoperfusion among the four distinct spatial patterns of WMHs, highlighting the need for subtype-specific pathomechanistic and therapeutic investigations.

## Data Availability

The raw data supporting the conclusions of this article will be made available by the authors, without undue reservation.
