# Supplementary material for: Vascular risk factors and neuroimaging heterogeneity across different white matter hyperintensities distribution patterns
Source: Front Hum Neurosci. 2025 Jul 28;19:1633355. doi: 10.3389/fnhum.2025.1633355 (PMC12336115; doi:10.3389/fnhum.2025.1633355)
Supplement: Supplementary file 1 [file Table_1.docx]

Supplementary Table 1: Association of different WMH patterns and MoCA scores

|  | MoCA | *p* value |
| --- | --- | --- |
| Multi-spots |  | 0.083 |
| Yes | 22.1±5.1 |  |
| No | 20.34±6.2 |  |
| Peri-BG |  | 0.618 |
| Yes | 21.2±6.3 |  |
| No | 21.8±5.1 |  |
| Anterior SC patches |  | 0.001^*^ |
| Yes | 19.7±5.7 |  |
| No | 22.7±5.0 |  |
| Posterior SC patches |  | 0.315 |
| Yes | 21.3±5.4 |  |
| No | 22.1±5.3 |  |

WMH, white matter hyperintensities; MoCA, Montreal Cognitive Assessment; BG, basal ganglia; SC, subcortical.

* *p* < 0.0125 after Bonferroni correction
